# Spatial and temporal analysis of African swine fever front-wave velocity in wild boar: implications for surveillance and control strategies

**DOI:** 10.3389/fvets.2024.1353983

**Published:** 2024-03-25

**Authors:** Marta Martínez Avilés, Fernando Montes, Irene Sacristán, Ana de la Torre, Irene Iglesias

**Affiliations:** ^1^Epidemiology and Environmental Health Group, Department of Infectious Animal Diseases and Global Health, Animal Health Research Centre, National Centre Institute for Agriculture and Food Research and Technology, Spanish National Research Council (CISA-INIA-CSIC), Madrid, Spain; ^2^Center for International Forestry Research, National Centre Institute for Agriculture and Food Research and Technology, Spanish National Research Council (CIFOR, INIA-CSIC), Madrid, Spain

**Keywords:** disease dynamics, spatial epidemiology, wild boar, velocity, ASF virus distribution, ASF virus spread, African swine fever

## Abstract

The front-wave velocity of African swine fever (ASF) virus spread is depicted through a retrospective spatial and temporal analyses of wild boar outbreaks from Jan. 2014 to Jan. 2022 in Estonia, Latvia, Lithuania and Eastern Poland—regions responsible for more than 50% of all wild boar cases in the EU. The study uses empirical semivariograms in a universal kriging model to assess spatial autocorrelation in notification dates and identifies a discernable large-scale spatial trend. The critical parameter of ASF front-wave velocity was identified (Mean = 66.33 km/month, SD = 163.24) in the whole study area, and explored the variations across countries, wild boar habitat suitability, seasons, and the study period. Statistical differences in front-wave velocity values among countries and temporal clusters are explored, shedding light on potential factors influencing ASF transmission dynamics. The implications of these findings for surveillance and control strategies are discussed.

## Introduction

Since the introduction of African swine fever (ASF) virus genotype II in the European Union (EU) in 2014, over 50,000 wild boar have succumbed to the virus, either hunted for surveillance or found dead (1). Initially confined to Estonia, Latvia, Lithuania and Poland from 2014 to 2016, the virus predominantly affected wild boar populations, constituting 97% of all notifications. Subsequently, in 2017, ASF reached the wild boar population of Czechia and was introduced in the domestic pig population of Romania. From 2018 onwards, ASF virus genotype II extended its spread to Belgium, Bulgaria, Germany, Greece, Hungary, Italy, and Slovakia within the EU. Notably, the virus’ spread intensified globally in 2018, reaching China, expanding rapidly across Asia, and extending to Oceania (Papua New Guinea in 2020) and the Caribbean countries of Dominican Republic and Haiti in 2021 (2).

ASF virus affects both wild and domestic pigs, primarily transmitting through direct or indirect contact with infected animals, their meat, or products, as well as materials or environments contaminated with the virus (3). Wild boar can contract ASF through feeding on infected carcasses or contaminated garbage, direct contact with other infectious wild boar or domestic pigs, or indirect contact with virus-contaminated environments (i.e., from infectious blood or carcasses) (3). ASF virus remains infectious in body parts and the environment for an extended period, especially in cold temperatures, with susceptible animals acquiring infection through nasal or oral contact with infected materials (products, body-parts, excretions or secretions of infectious animals) (3). While ticks of the *Ornithodoros* genus have historically transmitted and maintained the virus, their role in the current ASF epidemic in Europe is unknown (3).

Surveillance and control measures for ASF in wild boar in the EU encompass early detection, carcass removal, population management, including depopulation, and laboratory confirmation of virus presence or antibodies from biological samples of dead and hunted wild boar (4). The role of wild boar movements through natural corridors as a probable mechanism for introducing ASF into unaffected areas and facilitating its spread is well documented (5–11). Based on data extracted from the European Union Animal Disease Information System (12), ASF is present in wild boar populations in several EU countries (Germany, Czechia, Slovakia, Hungary, Romania, Bulgaria, and Italy, in addition to the Baltic countries and Poland, at the time of writing). Estonia, Latvia, Lithuania and Eastern Poland collectively account for more than 50% of all of wild boar cases in the EU. The median annual percentage of notifications in wild boar vs. domestic pigs in these four countries has been 99% and has never been below 80%.

Understanding the dynamics of ASF in wild boar populations is challenging due to limited data on population and movement patterns data, as well as logistical and cost constraints in surveillance efforts. Spatial modelling becomes crucial for anticipating the spread following confirmed ASF events in wild boar, identifying whether the spread is expected to be constant or increasing, or if further cases are expected in other areas and at which rate of occurrence, to implement targeted interventions. In this study, we hypothesize that the spatial dynamics of ASF virus in wild boar in the Baltic countries and Eastern Poland, where spill-over events at the domestic-wild boar interface have been infrequent along a 7-year period, exhibit varying rates of advancement. Such rates can be modelled by estimating the velocity of a front epidemic wave. In this study, we propose a kriging method to interpolate monthly ASF notifications in wild boar. This method provides a comprehensive analysis of space and time parameters within the context of ASF spread dynamics.

## Materials and methods

### Study area and data source

The study encompasses areas in Estonia, Latvia, Lithuania and Eastern Poland affected by ASF in wild boar from January 2014 until January 2022. We utilized ASF notifications reported to the EU Animal Diseases Information System (ADIS), which in addition to the species, provide details on the date of event confirmation, the number of cases per event, geographical coordinates where cases are found and any other epidemiologically relevant information as estimated by the reporting country. This additional information may include details such as whether the wild boar were found dead or shot, or the type of laboratory test employed for confirmation.

For each ASF notification in wild boar, we included information regarding the date and location. ASF notifications were mapped using the WGS84 projected coordinate system (ArcGIS Pro 3.0.3.) and depicted through a kernel density map.

### Geostatistical analysis of notifications in wild boar: the kriging model

The front-wave epidemic velocity has been estimated for other diseases, like bluetongue (13) and rabies (14), using trend surface analysis (TSA), a spatial interpolation technique used in geostatistics to estimate values at unobserved locations based on known values at sampled locations. Here, we use a kriging method for interpolating monthly ASF notifications in wild boar. Kriging is also a geostatistical stochastic technique of interpolation in which a linear combination of weights at known locations is used to estimate the value at unknown locations (15). Kriging explicitly models spatial correlation and is considered more robust than TSA when there is spatial dependence. It assumes that values closer in space tend to be more similar, and thus, the spatial correlation between sampled points is indicative of the correlation at unsampled locations. This way, kriging provides a more accurate representation of the variation within the variable (velocity) than what is achieved by TSA and the resulting prediction surface is more consistent with the input values (ASF notifications) than TSA (16). TSA is more often used when there is a known or hypothesized spatial trend that explains the observed variation in the notifications. Our approach avoids the need for conditioners or assumptions, thereby enabling a holistic investigation of spatial and temporal parameters within the framework of the dynamics of ASF propagation.

The study area was rasterized into 50km^2^ cells and the date of the earliest monthly ASF notification in wild boar was extracted for each cell.

Subsequently, a universal kriging model was applied to the earliest ASF monthly notifications in wild boar per cell throughout the study area. Under the universal kriging model the target variable at a given location *s_i_* is given by the sum of a trend function, which is a linear combination of p + 1 auxiliary variables *f*_j_(*s_i_*), multiplied by their respective beta coefficient, and a residual which shows spatial autocorrelation (17) (Eq. 1):


(1) $$Z\left(s_{i}\right)=\sum_{j=0}^{p+1}\beta_{j}f_{j}\left(s_{i}\right)+\delta \left(s_{i}\right)$$

Where, in our case, *Z*(*s_i_*) is the earliest ASF notification date at location *s_i_*, *f*_0_(*s_i_*) ≡ 1 ∇ *s_i_*, *f*_1_(*s_i_*) is the X coordinate, which was selected as the unique auxiliary variable standing for the spatial trend after evaluation of the *p*-value of the β-coefficients and one-leave-out cross-validation residuals RMSE and bias for the possible combinations of X, X^2^, Y and Y^2^, and δ (*s_i_*) is a residual the spatial autocorrelation of which is expressed by the semivariogram (Eq. 2):


(2) $$\gamma \left(h\right)=\frac{1}{2N\left(h\right)}\sum_{i=1}^{N\left(h\right)}{\left(\delta \left(s_{i}\right)-\delta \left(s_{i}+h\right)\right)}^{2}$$

Where γ (*h*) is the semivariance for the distance *h*, *N*(*h*) are the number of pair of cells with ASF notifications located at a distance *h* and δ(*s_i_*) – δ(*s_i_* + *h*) is the residual difference between two points separated by a distance *h*. In practice, γ (*h*) is averaged within determined distance lags. The semivariogram was modelled through a spherical semi-variogram following the description in Iglesias et al. (8). The Iterative Reweighted Least-Squares estimation (18) was used for the mean function coefficients and variogram parameters simultaneous estimation (19). Cross-validation was used to check the unbiasedness and kriging variance estimation accuracy of the model (20). The universal kriging prediction at location *s*_0_ was (Eq. 3):


$$p\left(Z,s_{0}\right)=\overset{n}{\underset{i=1}{\sum }}\lambda_{i}Z\left(s_{i}\right)$$

(3) $$\sum_{i=1}^{n}\lambda_{i}f_{j}\left(s_{i}\right)=f_{j}\left(s_{0}\right)\;j=0,1$$

The kriging weights *λ_i_* are determined based on the semivariogram model fitted to minimize the prediction error (18). p(*Z*,*s_0_*) is estimated for the pixels covering the study area to yield maps of the predicted date of ASF notifications.

The predicted kriging time was represented by monthly interval isochrones using ArcGIS Pro 3.0.3.

### Velocity of ASF spread

To quantify the ASF front-wave epidemic velocity, the surface of the prediction time resulting from the kriging analysis was included with the 3D Analyst toolbox of ArcGIS Pro as a vector of the map slope (magnitude), following the approach described by Moore et al. (14). The inverse of the slope surface corresponds to the front-wave velocity of ASF at each location within the study area. The relationship between velocity (V) and slope (Sp) is given by V = s/t and Sp = rise/s, where “s” is space and “t” is time. In this context, rise was defined as the predicted monthly ASF occurrence time, so rise = t (in months). Therefore, V can be calculated as 1/Sp. Consequently, larger values of Sp mean slower velocity (1/Sp) and larger time interval “t” at similar distance result in slower V as well. In other words, a higher slope value implies slower ASF diffusion, while a larger vector of time (lower slope) leads to faster velocity of diffusion.

The front-wave velocity of ASF spread in wild boar was expressed in km/month and its values were explored with descriptive statistics in Excel 2016 and in IBM SPSS Statistics v.29.0. The front-wave velocity values (median, minimum, maximum) per country were compared by season (winter: December, January, February; spring: March to May; summer: June to August; autumn: September to November), month and year. The variation of the front-wave velocity values was also compared by country in the different wild boar habitat suitability quality categories (from 1 to 6, being 1 the lowest and 6 the highest quality of wild boar habitat suitability) described in Bosch et al. (21) and available online.

Space–time cluster aggregations of velocity of ASF spread were additionally explored using retrospective seasonal and normal models (SaTScan v10.0; 22). These models require the inclusion of case data and temporal information of each case. Cluster selection was implemented based with varying temporal window of 3 to 6 months to capture seasonal differences. Evaluation of cluster performance was conducted based on ranked relative risk (RR), log-likelihood ratio (LLR) values and *p*-values obtained from Monte Carlo hypothesis testing (9,999 permutations).

## Results

### Geostatistical analysis of notifications in wild boar: the kriging model

**Figure 1:**
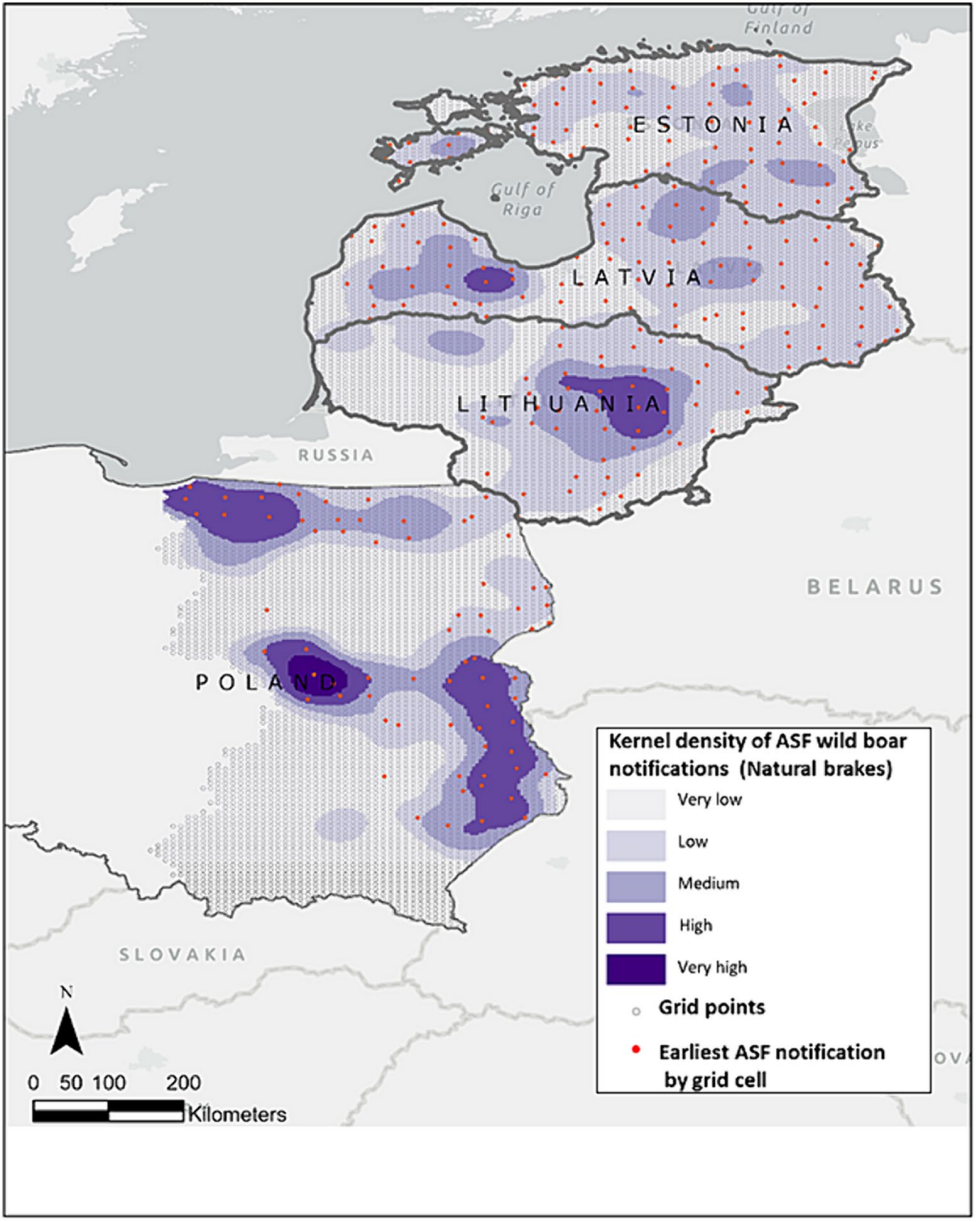
Study area (centroid of each cell grid) depicted in light green, overlaid is the kernel density of ASF wild boar notifications from January 2014 to January 2022, based on ADIS data shown in grey-blue shade. Red dots are the 291 earliest monthly ASF notifications in wild boar extracted for each cell grid, utilized as input in the kriging model.

The study area contains 9,693 grid cells of which 291 were selected for the universal kriging model, corresponding to 2,305 ASF wild boar notifications (Figure 1).

**Figure 2:**
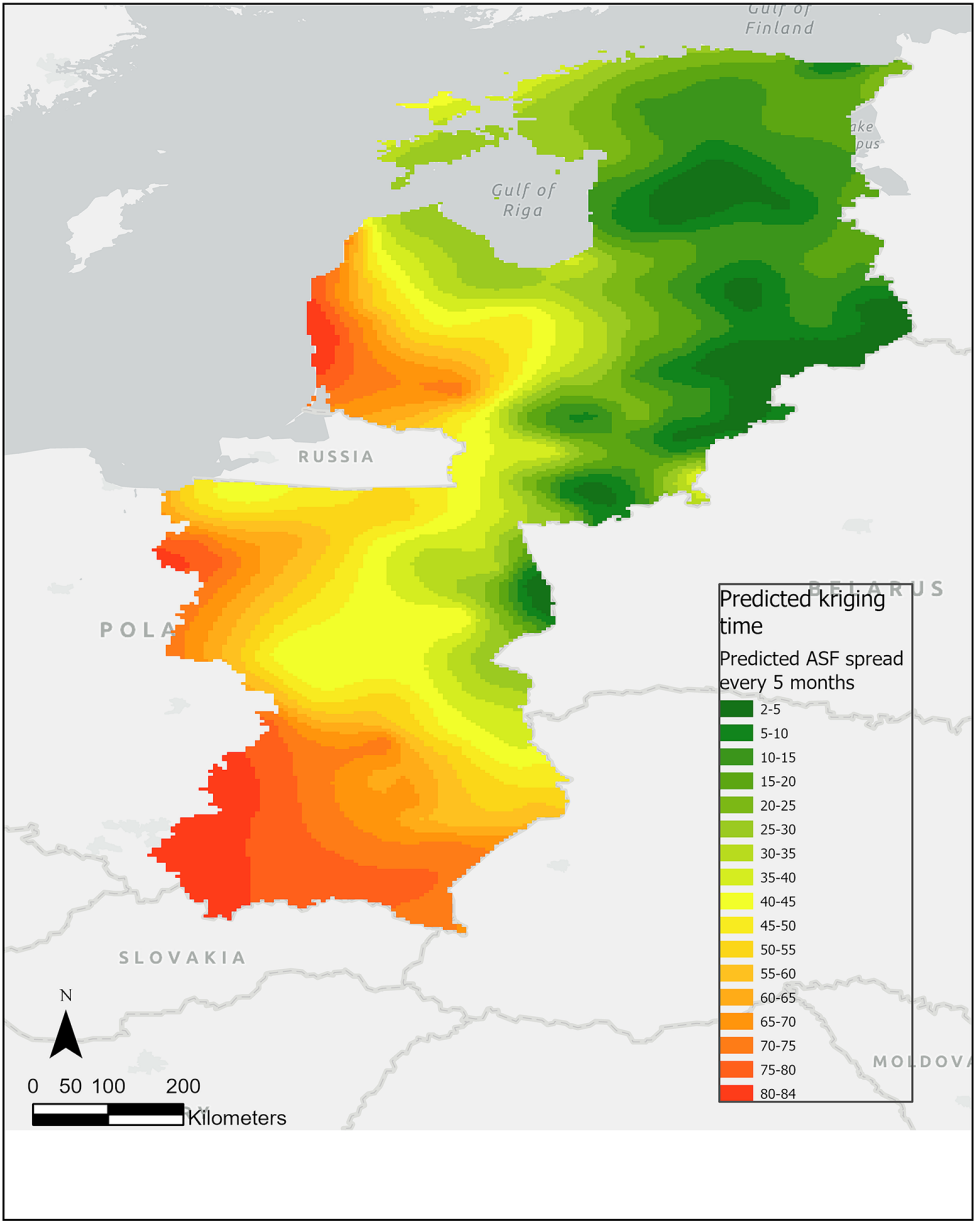
Predicted kriging time surface of ASF infection in wild boar at 5-month intervals from January 2014 to January 2022. In the choropleth map, the green color spectrum represents the early phases of ASF prediction events over time, while the red color spectrum represents the later months.

The empirical semivariogram shows strong spatial autocorrelation for notification dates and increasing large-scale semi-variance representing the spatial trend. Figure 2 shows the predicted kriging model fitted (γ) to the residual variogram and the linear combination γ + (β1 ∗ γ_X_)^2^ (where γ_X_ is the variogram of the X coordinate) fitted to the not detrended variogram. The one-leave-out cross-validation resulted in a bias of −0.43%, and a mean ratio of squared residuals/kriging variance of 1.0031.

### Velocity of spread of ASF in wild boar

**Figure 3:**
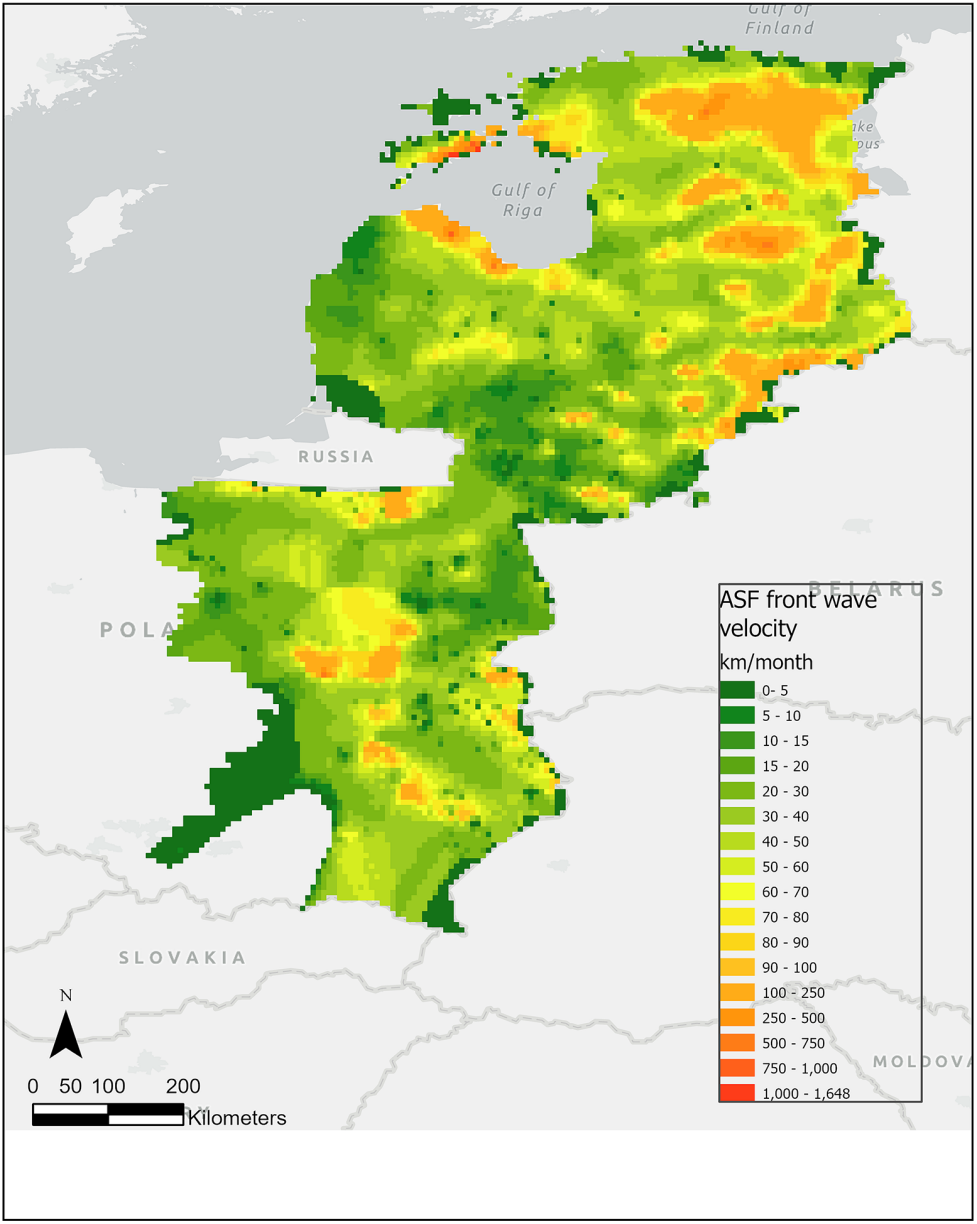
Predicted ASF front wave velocity in wild boar (km/month) during the period from January 2014 to January 2022. The green colors represent lower velocity, while the red colors represent higher velocity of the predicted ASF front-wave velocity in wild boar.

The front-wave velocity of ASF was obtained for 2,277 points with a median of 49.52 km/month (min.: 8.84; max.: 740.39) across the study area (Figure 3). By country, the median velocity was highest for Estonia (75.60 km/month (min.: 21.41; max.: 740.39), *n* = 397) and lowest for Lithuania [36.52 km/month (min.: 8.84; max.: 344.29), *n* = 258], followed by Poland [42.47 km/month (min.: 13.03; max.: 732.96), *n* = 1,179] and Latvia [53.95 km/month (min.: 11.98; max.: 697.05), *n* = 443].

By season, the highest median ASF front-wave velocity in Estonia and Latvia were obtained in autumn [Estonia: 90.28 km/month (min.: 22.9, max.: 740.39), *n* = 190; Latvia: 61.11 km/month (min.: 11.98; max.: 329.78), *n* = 183]. Interestingly, the second highest median in Estonia happened in winter [75.6 km/month (min.: 21.53, max.: 236.48), *n* = 105], while in Latvia it was in summer [59.79 km/month (min.: 16.45; max.: 697.05), *n* = 129]. However, the *n* was much smaller in Estonia in summer (*n* = 76) and in spring (*n* = 25). Similarly, the number of points in Latvia in spring was only 19. Curiously, the maximum velocities were reached in summer for both countries (640.21 km/month in Estonia and 697.05 km/month in Latvia). In Lithuania, the number of predicted points was fewer than for the other countries (*n* = 258 in total), but the distribution is even among the seasons (although spring has, again, the fewest prediction points). Here, the highest median front-wave velocity was achieved in summer (51.44 km/month) but the highest maximum was in autumn (344.23 km/month). Finally, in Eastern Poland, the number of predicted points was much higher in winter (*n* = 688) than in the other seasons (between 113 and 198). However, the velocity median was homogeneous across seasons (between 35.45 and 48.34 km/month) although the maximum was reached in winter (732.96 km/month).

The results by month shed similar conclusions. Generally, there is a sufficient number of points to allow comparison for the months between August and January (>10% of the total predicted points per country), but there are fewer points predicted from February to July (<5%), and too few in April, May and June (<2%) to be considered in the comparative descriptive analysis. The maximum velocities were reached in October and November in Estonia (706.36 and 740.39 km/month respectively), in Poland in February (732.96 km/month), and in Latvia in August (697.06 km/month).

The highest median predicted velocity per year occurred in Estonia in 2015 (89.30 km/month) and in Latvia in 2014 (81.69 km/month). The highest maximum velocity in Estonia was predicted in 2016 (740.39 km/month), in Latvia in 2015 (697.05 km/month), in Poland in 2018 (732.96 km/month) and in 2014 in Lithuania (344.29 km/month).

Similar results were obtained with SaTScan analysis, showing one higher velocity temporal cluster from August to November (Mean = 93.64, SD = 111.56; *p* = 0.001).

Finally, the majority of the predicted values (between 73 and 88%) fell in the maximum quality categories (5 and 6) of available habitat for wild boar, making comparisons among the rest of the categories at risk of misinterpretation due to sampling bias.

The complete output from the descriptive analysis can be found as Supplementary material S1.

## Discussion

The observed spatial autocorrelation in ASF wild boar notification dates indicates a non-random distribution over the 7-year period from 2014 to 2022 in the study area encompassing Estonia, Latvia, Lithuania and Eastern Poland. Kriging analysis facilitated a smoothed evaluation over time, enabling the description and visualization of the spread of ASF virus in wild boar in the study area. The identification of a large-scale spatial trend underscores the need to consider additional factors influencing ASF dynamics. Since the introduction of the ASF virus into the EU in 2014, its spread has occurred through two main pathways: wild boar-mediated (direct or indirect contact with an infectious wild boar, whether live or dead, or their excretions or secretions), and human-mediated (23). The latter includes trade or translocations, as well as any other activity involving humans that could transmit the virus in the environment, such as those related with pig farming or with wild boar hunting (24). While our study does not differentiate between transmission pathways, the significant differences in front-wave velocity values among countries, years and seasons highlight the heterogeneous nature of ASF transmission dynamics. The highest velocity in Estonia and the lowest in Lithuania could be indicating that local factors, such as biosecurity measures, pig farming practices, or environmental conditions, are contributing to variations in disease spread. ASF severely decimated the Estonian wild boar population from 2016 to 2020, an indication of the impact of the disease in this country (25).

The link between a seasonal pattern of ASF and wild boar disease dynamics has been previously observed in the Baltic States and Poland (26), but is highly biased by the surveillance efforts. Winter temperatures favour ASF virus infectiousness in tissues (27) and the preservation of carcasses for longer. However, higher interactions of wild boar with their deceased counterparts were observed at warmer temperatures when maggots and insects were present in the decomposing carcasses (28, 29). The identification of a temporal cluster from August to November could indicate a potential period of increased transmission risk, but should be interpreted with caution due to potential sampling bias.

The highest velocity in 2016 and the lowest in 2020 may be influenced by the control measures implemented during these years, as well as differences in surveillance efforts. In Estonia and Lithuania, the number of samples from hunted wild boar was highest in 2016, and after a lower number of samples in the following years, the number of samples increased again in 2021 (25). In all three Baltic countries the number of samples from passive surveillance was highest from 2014 to 2016 (25). The slower dynamic of ASF from 2017 to 2020 has also been noted in Poland by Bocian et al. (30).

The effect of habitat on wild boar movement was reviewed by Morelle et al. (31). The quality of habitat is influenced by the distribution and abundance of food resources, and wild boar movements seem to increase particularly under high population density and low food availability. Further investigation into the relationship between habitat characteristics and ASF transmission dynamics may provide valuable insights.

The velocity of ASF spread identified here allows us to explain the progress of the disease as an event. The intensity of this event is not defined here. For this, other parameters such as the number of affected animals or serology data should be included. For the purposes of policy and prevention practices, the economic consequences could be considered similar, as the notification of the disease, in addition to direct losses, leads to indirect losses derived from the “news effect” of the disease’s presence (for example, in Belgium ASF was only notified in wild boar, but domestic pig trade decreased in some sectors). Therefore, the identification of a velocity front is a sufficient parameter to initiate the evaluation of consequences derived from the arrival of the virus.

It is important to recognize that our findings may be influenced by bias and uncertainty inherent to the kriging methodology. Kriging, a method that explicitly captures spatial correlation, operates under the assumption that values in close proximity tend to exhibit greater similarity. This allows for the inference of correlation at locations that have not been sampled, based on the points that have been sampled (20). Even though kriging offers certain benefits compared to TSA in terms of capturing spatial dependence and variation, it is still susceptible to potential biases. Factors such as the quality and quantity of input data, particularly in regions with limited data, which can occur in disease notifications in wild animals whose surveillance is subject to fluctuations, can influence the accuracy of kriging predictions. Additionally, due to the possible delay between disease progression and notifications, and the interaction between the grid size (which was chosen considering the spatial distribution of notifications) and potential jumps in the advancing front, overestimations of the advancement speed may occur. Therefore, it is prudent to interpret our findings with caution, recognizing the inherent uncertainties associated with the methodology employed.

Understanding the spatial and temporal dynamics of ASF front-wave velocity is crucial for designing effective surveillance and control strategies. Tailoring interventions based on country-specific patterns, seasonal variations, and temporal clusters can enhance the efficiency of control measures and resource allocation.

## Data availability statement

The original contributions presented in the study are included in the article/Supplementary material, further inquiries can be directed to the corresponding author.

## Author contributions

MM: Data curation, Funding acquisition, Investigation, Resources, Software, Validation, Visualization, Writing – original draft. FM: Formal analysis, Methodology, Software, Writing – original draft. IS: Formal analysis, Investigation, Software, Writing – original draft. AT: Data curation, Investigation, Project administration, Resources, Supervision, Writing – review & editing. II: Conceptualization, Formal analysis, Investigation, Methodology, Resources, Software, Supervision, Validation, Writing – original draft.
