# Supplementary material for: Spatial and temporal analysis of African swine fever front-wave velocity in wild boar: implications for surveillance and control strategies
Source: Front Vet Sci. 2024 Mar 25;11:1353983. doi: 10.3389/fvets.2024.1353983 (PMC11002761; doi:10.3389/fvets.2024.1353983)
Supplement: Supplementary file 1 [file Table_1.DOCX]

Supplementary Material of the manuscript: “Spatial and temporal analysis of African swine fever front-wave velocity in wild boar: implications for surveillance and control strategies” by Martínez Avilés et al., submitted to Frontiers in Veterinary Science

Descriptive statistics of the front-wave ASF velocity in wild boar by country (in km/month) and variable of study (season, month, year, and quality of available habitat (QAH, see main text for reference)).

| ESTONIA | | | | |
| --- | --- | --- | --- | --- |
|  | **N** | **Median** | **Minimum** | **Maximum** |
| SEASON |  |  |  |  |
| Autumn | 190 | 90.2847 | 22.90 | 740.39 |
| Spring | 25 | 30.6568 | 21.41 | 112.44 |
| Summer | 76 | 57.1782 | 21.41 | 640.21 |
| Winter | 106 | 66.0682 | 21.53 | 236.48 |
| MONTH |  |  |  |  |
| January | 40 | 49.5206 | 25.24 | 234.61 |
| February | 18 | 41.5498 | 21.53 | 117.68 |
| March | 14 | 26.4296 | 21.53 | 89.29 |
| April | 3 | 33.8553 | 21.41 | 38.59 |
| May | 8 | 40.0067 | 22.59 | 112.44 |
| June | 7 | 53.7846 | 22.13 | 56.57 |
| July | 23 | 50.0185 | 21.41 | 383.16 |
| August | 46 | 71.8692 | 30.07 | 640.21 |
| September | 60 | 102.9635 | 27.39 | 328.17 |
| October | 58 | 92.8010 | 27.27 | 706.36 |
| November | 72 | 78.2587 | 22.90 | 740.39 |
| December | 48 | 80.0142 | 33.38 | 236.48 |
| YEAR |  |  |  |  |
| 2014 | 34 | 49.5206 | 48.85 | 132.29 |
| 2015 | 213 | 89.3054 | 21.41 | 383.16 |
| 2016 | 109 | 69.0902 | 22.90 | 740.39 |
| 2017 | 38 | 27.5647 | 21.53 | 88.22 |
| 2018 |  |  |  |  |
| 2019 | 3 | 46.8275 | 43.56 | 122.28 |
| 2020 |  |  |  |  |
| 2021 |  |  |  |  |
| QAH CATEGORIES |  |  |  |  |
| 0 | 1 |  |  |  |
| 1 | 1 |  |  |  |
| 2 |  |  |  |  |
| 3 | 24 | 76.5139 | 34.79 | 236.48 |
| 4 | 20 | 43.8065 | 22.59 | 158.49 |
| 5 | 49 | 83.8319 | 27.27 | 383.16 |
| 6 | 302 | 72.1294 | 21.41 | 740.39 |

| LATVIA | | | | |
| --- | --- | --- | --- | --- |
|  | **N** | **Median** | **Minimum** | **Maximum** |
| SEASON |  |  |  |  |
| Autumn | 183 | 61.1096 | 11.98 | 329.78 |
| Spring | 19 | 27.4079 | 12.92 | 54.25 |
| Summer | 129 | 59.7866 | 16.45 | 697.05 |
| Winter | 112 | 40.9823 | 12.57 | 382.61 |
| MONTH |  |  |  |  |
| January | 44 | 46.5540 | 16.31 | 382.61 |
| February | 10 | 54.2817 | 15.75 | 163.79 |
| March | 9 | 27.9771 | 12.92 | 44.91 |
| April | 5 | 16.4492 | 15.11 | 54.25 |
| May | 5 | 44.9067 | 19.63 | 51.99 |
| June | 9 | 38.3924 | 17.57 | 59.51 |
| July | 52 | 61.0471 | 16.45 | 326.21 |
| August | 68 | 65.0329 | 32.86 | 697.05 |
| September | 59 | 59.5052 | 18.98 | 329.78 |
| October | 62 | 64.9937 | 15.26 | 289.40 |
| November | 62 | 51.6919 | 11.98 | 210.20 |
| December | 58 | 38.9415 | 12.57 | 136.68 |
| YEAR |  |  |  |  |
| 2014 | 116 | 81.6939 | 31.10 | 326.21 |
| 2015 | 127 | 46.6462 | 23.56 | 697.05 |
| 2016 | 101 | 67.9317 | 16.45 | 289.40 |
| 2017 | 59 | 37.1120 | 11.98 | 170.99 |
| 2018 | 23 | 19.0409 | 12.92 | 41.98 |
| 2019 | 13 | 64.0145 | 15.26 | 382.61 |
| 2020 | 4 | 17.3016 | 15.75 | 19.24 |
| 2021 |  |  |  |  |
| QAH CATEGORIES |  |  |  |  |
| 0 |  |  |  |  |
| 1 |  |  |  |  |
| 2 |  |  |  |  |
| 3 | 41 | 47.1305 | 17.57 | 326.21 |
| 4 | 25 | 46.6462 | 11.98 | 364.31 |
| 5 | 52 | 54.4112 | 16.31 | 329.78 |
| 6 | 325 | 55.7982 | 12.57 | 697.05 |

| LITHUANIA | | | | |
| --- | --- | --- | --- | --- |
|  | **N** | **Median** | **Minimum** | **Maximum** |
| SEASON |  |  |  |  |
| Autumn | 57 | 38.0875 | 11.00 | 344.29 |
| Spring | 30 | 33.4141 | 15.24 | 50.54 |
| Summer | 84 | 51.4361 | 8.84 | 122.70 |
| Winter | 87 | 22.3144 | 11.44 | 138.45 |
| MONTH |  |  |  |  |
| January | 40 | 19.7742 | 11.45 | 131.77 |
| February | 11 | 19.7742 | 15.24 | 103.13 |
| March | 13 | 43.7166 | 15.24 | 45.41 |
| April | 9 | 22.9941 | 15.86 | 41.11 |
| May | 8 | 33.4141 | 21.21 | 50.54 |
| June | 14 | 28.8667 | 8.84 | 76.75 |
| July | 49 | 57.1292 | 12.95 | 84.56 |
| August | 21 | 47.2553 | 22.79 | 122.70 |
| September | 12 | 45.8414 | 16.70 | 129.31 |
| October | 16 | 53.4636 | 14.86 | 201.12 |
| November | 29 | 25.1919 | 11.00 | 344.29 |
| December | 36 | 26.3112 | 11.44 | 138.45 |
| YEAR |  |  |  |  |
| 2014 | 37 | 65.4717 | 23.18 | 344.29 |
| 2015 | 35 | 31.1833 | 18.86 | 175.58 |
| 2016 | 7 | 13.7701 | 11.44 | 28.92 |
| 2017 | 47 | 19.7742 | 11.00 | 56.46 |
| 2018 | 120 | 41.6422 | 11.45 | 84.56 |
| 2019 | 4 | 20.8100 | 8.84 | 26.54 |
| 2020 | 8 | 23.0914 | 19.71 | 24.83 |
| 2021 |  |  |  |  |
| QAH CATEGORIES |  |  |  |  |
| 0 | 2 |  |  |  |
| 1 | 1 |  |  |  |
| 2 |  |  |  |  |
| 3 | 26 | 36.8106 | 12.2 | 344.29 |
| 4 | 7 | 35.6513 | 19.72 | 131.77 |
| 5 | 31 | 35.4037 | 8.84 | 74.84 |
| 6 | 191 | 35.4178 | 11 | 201.12 |

| EASTERN POLAND | | | | |
| --- | --- | --- | --- | --- |
|  | **N** | **Median** | **Minimum** | **Maximum** |
| SEASON |  |  |  |  |
| Autumn | 198 | 42.6566 | 16.73 | 181.01 |
| Spring | 113 | 35.4502 | 14.85 | 143.23 |
| Summer | 180 | 39.8618 | 14.94 | 120.95 |
| Winter | 688 | 48.3486 | 13.03 | 732.96 |
| MONTH |  |  |  |  |
| January | 336 | 40.7591 | 14.14 | 314.11 |
| February | 180 | 47.2404 | 14.02 | 732.96 |
| March | 74 | 39.3895 | 14.94 | 143.23 |
| April | 22 | 35.5792 | 14.85 | 58.39 |
| May | 17 | 26.9488 | 15.01 | 72.63 |
| June | 15 | 44.6310 | 20.10 | 79.60 |
| July | 75 | 37.9883 | 14.94 | 120.95 |
| August | 90 | 41.6152 | 16.73 | 117.51 |
| September | 47 | 40.6194 | 17.21 | 101.67 |
| October | 32 | 39.8865 | 16.73 | 117.51 |
| November | 119 | 52.8902 | 17.21 | 181.01 |
| December | 172 | 59.7494 | 13.03 | 367.59 |
| YEAR |  |  |  |  |
| 2014 | 6 | 20.0994 | 13.56 | 29.78 |
| 2015 | 7 | 15.3943 | 13.03 | 20.18 |
| 2016 | 43 | 65.4537 | 21.37 | 367.59 |
| 2017 | 275 | 59.7494 | 19.10 | 196.49 |
| 2018 | 444 | 59.7494 | 16.87 | 732.96 |
| 2019 | 129 | 36.4712 | 14.94 | 117.30 |
| 2020 | 242 | 28.2645 | 14.14 | 88.45 |
| 2021 | 33 | 34.9238 | 22.89 | 77.53 |
| QAH CATEGORIES |  |  |  |  |
| 0 | 31 |  |  |  |
| 1 | 4 |  |  |  |
| 2 |  |  |  |  |
| 3 | 226 | 47.2404 | 15.01 | 367.59 |
| 4 | 57 | 40.7965 | 16.05 | 144.14 |
| 5 | 265 | 48.6429 | 13.03 | 314.11 |
| 6 | 596 | 39.583 | 13.56 | 732.96 |
